# Associations of artificial intelligence use at work with psychological distress, loneliness, and life satisfaction among employed US adults

**DOI:** 10.1016/j.pmedr.2026.103640

**Published:** 2026-09-18

**Authors:** Jemar R. Bather, Adolfo G. Cuevas, Melody S. Goodman, José A. Pagán

**Affiliations:** a Department of Biostatistics, NYU School of Global Public Health, New York, NY, USA; b Department of Social and Behavioral Sciences, NYU School of Global Public Health, New York, NY, USA; c Department of Public Health Policy and Management, NYU School of Global Public Health, New York, NY, USA

**Keywords:** Generative artificial intelligence, ChatGPT, Copilot, Workplace automation, Task automation, Mental distress, Depression, Anxiety, Subjective well-being, American workers

## Abstract

**Objective:**

To quantify the associations between artificial intelligence use at work and psychosocial well-being among employed adults in the United States.

**Methods:**

Nationally representative data come from the Household Trends and Outlook Pulse Survey (United States, March 2026). Artificial intelligence use at work was measured as the number of distinct assisted work tasks. Outcomes included psychological distress, loneliness, and life satisfaction. Regression models adjusted for sociodemographic characteristics, region of residence, occupation, and telework frequency.

**Results:**

The data generalized to 152 million employed U.S. adults, with 51.7% using artificial intelligence at work. The most common artificial intelligence task was “searching for information or technical help” (35.8%), followed by “writing communications, documentation, or instructions” (31.6%). Greater artificial intelligence use at work was associated with greater life satisfaction (0.11 SD increase, 95% CI: 0.06, 0.16) and a higher prevalence of feeling lonely (aPR: 1.13, 95% CI: 1.02, 1.25). There was no significant association with moderate-to-severe psychological distress.

**Conclusions:**

The findings provide evidence that artificial intelligence use at work is associated with life satisfaction and loneliness, but not with overall psychological distress. Longitudinal studies are needed to examine the impact of increasing artificial intelligence use at work on employee psychosocial well-being.

## Highlights

• Greater artificial intelligence use at work was associated with higher life satisfaction.
• Artificial intelligence use at work was associated with greater prevalence of loneliness.
• Artificial intelligence use at work was not associated with psychological distress.

## 1 Introduction

Artificial intelligence tools, including large language models, have become part of everyday use in the United States (U.S.) and abroad (Bick et al., 2026; P. Song et al., 2026). Recent U.S. data show that one in four adults use an artificial intelligence tool or chatbot for health information or advice (Raynes and Maese, 2026). Despite the growing use of artificial intelligence to seek health information, few studies have examined the relationship between artificial intelligence use and psychosocial well-being (Perlis et al., 2026a, Perlis et al., 2026b). One nationally representative study found that greater artificial intelligence use was associated with adverse mental health symptoms, with about half of daily artificial intelligence users reporting using it for work alongside personal use (Perlis et al., 2026b). Building on this foundation, an open question is whether these associations extend to artificial intelligence use embedded in paid work, and whether they generalize beyond a single mental health outcome to a broader range of psychosocial states that workplace technology could plausibly affect.

Several distinct mechanisms may link workplace artificial intelligence use to psychosocial well-being, and these pathways could plausibly operate in different directions. First, artificial intelligence assistance with tasks such as information retrieval or communication drafting may reduce the time and effort required for routine components of work, potentially enhancing worker productivity and short-term job satisfaction (Brynjolfsson et al., 2025; Noy and Zhang, 2023). Second, the same reduction in routine tasks may reduce the casual, low-stakes interactions that occur when coworkers consult each other for information or feedback. In line with this, greater frequency of artificial intelligence interaction at work has been associated with increased loneliness, a distinct maladaptive response documented alongside, but not mediated by, an adaptive rise in the need for affiliation (Tang et al., 2023). Third, artificial intelligence adoption may extend the length of the workday (Jiang et al., 2025), while work pace accelerates and workers face added cognitive strain from integrating a new tool in their workflows. This could heighten psychological distress through work intensification and fatigue, even as output improves (Ranganathan and Ye, 2026). Collectively, these mechanisms indicate that workplace artificial intelligence use may relate to different psychosocial well-being outcomes in varying directions, which motivates examining conceptually distinct outcomes rather than a single, global mental health measure.

Building on this work, the present study focused on the extent of artificial intelligence use at work, using nationally representative data on employed U.S. adults to assess its association with psychological well-being outcomes. Specifically, we examined whether the extent of artificial intelligence use at work was significantly related to moderate-to-severe psychological distress, loneliness, and life satisfaction, after adjusting for demographic characteristics, socioeconomic status (SES), region of residence, occupation type, and telework frequency.

## 2 Methods

### 2.1 Study design and population

This study used nationally representative data from the Household Trends and Outlook Pulse Survey (HTOPS), fielded from March 13 to March 30, 2026, to measure emergent social and economic issues impacting U.S. households (US Census Bureau, 2026). Households were randomly sampled, with one individual per household invited to participate. This study followed the Strengthening the Reporting of Observational Studies in Epidemiology guidelines for cross-sectional studies (von Elm et al., 2007).

A total of 12,521 U.S. adults participated in HTOPS from March 13 to March 30, 2026. A total of 5693 (45.5%) individuals who were unemployed, 148 (1.2%) participants with missing employment status data, and 442 (3.5%) participants with missing outcome data were excluded from the analysis. The final analytic sample comprised 6238 employed U.S. adults.

### 2.2 Measures

The outcomes of interest were psychological distress, loneliness, and life satisfaction. The Patient Health Questionnaire-4 measured psychological distress (Kroenke et al., 2009). Using a four-point scale from *0 (not at all)* to *3 (nearly every day)*, participants rated how often in the past two weeks they were (a) feeling nervous, anxious, or on edge, (b) not being able to stop or control worrying, (c) having little interest or pleasure in doing things, and (d) feeling down, depressed, or hopeless. Responses were summed (Cronbach's α = 0.86) and then dichotomized into moderate-to-severe psychological distress (scores 6 to 12) and no-significant-to-mild psychological distress (scores 0 to 5) (Kroenke et al., 2009). Loneliness was assessed using one item: “How often do you feel lonely?” Response options were classified as always/usually/sometimes and rarely/never (Bruss et al., 2024). Life satisfaction was measured using a 10-point response scale from *0 (not satisfied at all)* to *10 (completely satisfied)* as the answer to the following question: “Overall how satisfied are you with life as a whole these days?” Life satisfaction was standardized to have mean = 0 and standard deviation (SD) = 1.

Extent of artificial intelligence use at work was measured using 11 items. Participants were shown the following prompt: “Artificial intelligence (AI) is the capability of computational systems to perform tasks typically associated with human intelligence, such as learning, reasoning, problem-solving, perception, and decision-making. (Examples of AI: machine learning, natural language processing, virtual agents, voice recognition, etc.)” The survey then asked: “In your work, have you used AI to do any of the following tasks? Select Yes or No for each.” The options included (1) Administrative tasks, (2) Coding or modeling, (3) Customer support, (4) Data analysis and visualization, (5) Generating ideas, (6) Interpreting, translating, or summarizing, (7) Managing logistics or supply chains, (8) Medical care, (9) Searching for information or technical help, (10) Tutoring, training, or educational assistance, and (11) Writing communications, documentation, or instructions. A composite score was derived as the sum of affirmative responses (range 0 to 11) and standardized to have mean = 0 and SD = 1.

Covariates were selected based on prior research (Beckel and Fisher, 2022; Bick et al., 2026; He et al., 2026; Henke et al., 2016; Islam et al., 2023; Leong et al., 2025; Song and Gao, 2020). Age was measured continuously in years. Sex was recorded as male or female. Racial/ethnic groups included Hispanic (any race), White, Black, Asian, and Other. Marital status was grouped as married, divorced/separated/widowed, or never married. Educational attainment was classified as high school or less, some college/associate's degree, bachelor's degree, or graduate degree. Annual household income was categorized as less than $50,000, $50,000 to $99,999, $100,000 to $149,999, or $150,000 and above. Census region of residence was classified as Northeast, Midwest, South, or West. Occupation types included: (1) Computer occupations (for example, computer support specialists, software developers, data scientists), (2) Construction and skilled trades, production, transportation, and material moving occupations, (3) Education and social services and arts, design, entertainment, and media occupations, (4) Engineering, architecture, and life, physical, and social sciences occupations, (5) Farming, fishing, and forestry, (6) Healthcare practitioners and healthcare support occupations, (7) Management, business operations, financial, and legal occupations, (8) Office and administrative support occupations, (9) Sales occupations, (10) Security, safety, food, cleaning, groundskeeping, and personal care services occupations. Due to a small unweighted sample size (*n* = 21), farming/fishing/forestry was combined with construction and skilled trades, production, transportation, and material moving occupations to create a “Trades, Transport, and Farming” group. Telework frequency was measured as no days, 1 to 2 days, 3 to 4 days, or 5+ days.

### 2.3 Statistical analysis

Summary statistics are presented using unweighted counts with weighted proportions for categorical measures or as weighted means with weighted standard errors (SEs) for continuous measures. The design-based Kruskal-Wallis test and the chi-squared test with the Rao and Scott adjustment were used to evaluate whether characteristics differed significantly between those who used artificial intelligence for work and those who did not. Little's Missing Completely at Random test was statistically significant ($\chi_{62}^{2}$ = 163.79, *p* < 0.001), indicating that missing covariate data were not missing completely at random (Little, 1988). Accordingly, multivariate imputation by chained equations under the missing at random assumption was employed via the *mice* R package (Buuren & van Buuren and Groothuis-Oudshoorn, 2011), generating ten imputed datasets that were analyzed separately and pooled using Rubin's (1987) rules.

We employed multivariable regression to estimate the association between extent of artificial intelligence use at work and psychosocial well-being (Hidalgo and Goodman, 2013). Robust Poisson regression was specified for psychological distress and loneliness models (Barros and Hirakata, 2003). Linear regression was specified for life satisfaction models. All models used the same sequential covariate adjustment strategy. Model 1 regressed the outcome on the standardized count of artificial intelligence tasks. Model 2 additionally adjusted for demographic characteristics (age, sex, race/ethnicity, and marital status). Model 3 additionally adjusted for SES (educational attainment, household income) and region of residence. Model 4 further adjusted for occupation type and telework frequency. The complex survey design was accounted for through the *survey* R package (Lumley, 2004). All analyses were conducted using R version 4.5.2 (R Project for Statistical Computing), with *p* < 0.05 considered statistically significant. Institutional Review Board review was not required for this secondary data analysis, as the data are publicly available in de-identified form on the U.S. Census Bureau website (US Census Bureau, 2026).

## 3 Results

### 3.1 Weighted univariate statistics

The data generalized to nearly 152 million employed U.S. adults (mean age = 42.2, SE = 0.3), with 50.5% male, 59.6% White, and 58.8% married (Table 1). About a third (33.8%) had a high school education or less, 31.5% had an annual household income between $50,000 and $99,999, and 38.7% resided in the South. The top three occupation types were: trades/transport/farming (19.2%), management/business/legal (16.0%), and healthcare (15.6%). Regarding the outcomes, the prevalence for symptoms of moderate-to-severe psychological distress was 7.5%, and the prevalence for loneliness was 26.4%. The mean life satisfaction score was 6.9 (SE = 0.1). A little over half of employed U.S. adults (51.7%, 95% CI: 47.6%, 55.9%) used artificial intelligence at work. Life satisfaction was negatively correlated with moderate-to-severe psychological distress (*r* = −0.46, 95% CI: −0.53, −0.39) and loneliness (*r* = −0.36, 95% CI: −0.43, −0.29; Supplemental Table 1).

**Table 1:** Sample characteristics, overall and by artificial intelligence use at work among employed U.S. adults, 2026 Household Trends and Outlook Pulse Survey. Counts are unweighted (unless specified as weighted). Proportions, means, and standard errors are weighted.

Characteristic	Overall	No artificial intelligence use	Any artificial intelligence use	*p*-value
N (unweighted)	6238	2369	3869	
N (weighted)	151,990,638	73,357,518	78,633,120	
Age, Mean (SE)	42.2 (0.3)	42.5 (0.7)	41.9 (0.6)	0.95
Sex, n (%)				0.57
Male	3390 (50.5)	1189 (49.4)	2201 (51.5)	
Female	2848 (49.5)	1180 (50.6)	1668 (48.5)	
Race/ethnicity, n (%)				0.20
White	3860 (59.6)	1440 (59.5)	2420 (59.8)	
Hispanic	663 (13.8)	307 (15.1)	356 (12.5)	
Black	558 (9.9)	260 (10.6)	298 (9.3)	
Asian	706 (8.0)	203 (5.8)	503 (10.1)	
Other	451 (8.6)	159 (9.0)	292 (8.3)	
Marital status, n (%)				<0.001
Married	3885 (58.8)	1314 (50.7)	2571 (66.3)	
Divorced/Separated/Widowed	1040 (10.0)	494 (12.4)	546 (7.8)	
Never married	1285 (31.2)	549 (36.9)	736 (26.0)	
Missing	28 (0.2)	12 (0.2)	16 (0.2)	
Educational attainment, n (%)				<0.001
High school or less	757 (33.8)	553 (48.6)	204 (20.0)	
Some college/Associate's degree	1383 (21.7)	708 (25.6)	675 (18.1)	
Bachelor's degree	2017 (24.0)	654 (16.5)	1363 (30.9)	
Graduate degree	2081 (20.5)	454 (9.3)	1627 (31.0)	
Household income, n (%)				<0.001
Less than $50,000	753 (19.0)	462 (27.8)	291 (10.6)	
$50,000–$99,999	1383 (31.5)	717 (36.9)	666 (26.4)	
$100,000–$149,999	1191 (19.8)	454 (18.6)	737 (20.9)	
$150,000 and above	2436 (29.7)	533 (16.8)	1903 (42.1)	
Missing	475 (9.5)	203 (8.1)	272 (10.8)	
Census region, n (%)				0.012
Northeast	1206 (16.6)	464 (15.5)	742 (17.7)	
Midwest	1000 (21.6)	442 (26.0)	558 (17.5)	
South	2740 (38.7)	982 (34.8)	1758 (42.2)	
West	1292 (23.1)	481 (23.8)	811 (22.5)	
Occupation, n (%)				<0.001
Computer	607 (6.9)	70 (1.4)	537 (11.8)	
Trades, Transport, and Farming	598 (19.2)	424 (31.5)	174 (8.5)	
Education/Social Services/Arts	802 (11.7)	241 (7.4)	561 (15.4)	
Engineering/Architecture/Sciences	427 (5.0)	105 (2.5)	322 (7.3)	
Healthcare	839 (15.6)	363 (16.9)	476 (14.4)	
Management/Business/Legal	1396 (16.0)	338 (9.4)	1058 (21.7)	
Office/Administrative Support	512 (7.3)	260 (7.1)	252 (7.4)	
Sales	467 (8.5)	165 (8.0)	302 (8.9)	
Services/Security/Food/Personal Care	400 (9.9)	279 (15.9)	121 (4.6)	
Missing	190 (5.7)	124 (8.8)	66 (2.7)	
Telework frequency, n (%)				<0.001
No telework	3461 (70.9)	1789 (88.0)	1672 (54.9)	
1–2 days	1102 (12.4)	220 (4.6)	882 (19.7)	
3–4 days	555 (5.4)	120 (2.1)	435 (8.5)	
5+ days	1094 (11.3)	233 (5.3)	861 (16.9)	
Missing	26 (0.2)	7 (0.1)	19 (0.4)	
Moderate-to-severe psychological distress, n (%)	450 (7.5)	177 (7.3)	273 (7.7)	0.71
Loneliness (Always/Usually/Sometimes), n (%)	1434 (26.4)	527 (24.8)	907 (28.0)	0.42
Life satisfaction, Mean (SE)	6.9 (0.1)	6.7 (0.1)	7.1 (0.1)	0.010

*p*-values: design-based Rao-Scott chi-squared (categorical) or Kruskal-Wallis (continuous), based on successive difference replication weights.

### 3.2 Weighted bivariate statistics

Artificial intelligence use at work significantly differed across marital status, educational attainment, household income, census region of residence, occupation type, telework frequency, and life satisfaction (Table 1). Compared to those who did not use artificial intelligence at work, those who did were more likely to be married (66.3% vs. 50.7%, *p* < 0.001), have a graduate degree (31.0% vs. 9.3%, *p* < 0.001), have a household income of $150,000 or more (42.1% vs. 16.8%, *p* < 0.001), live in the South (42.2% vs. 34.8%, *p* = 0.012), work in a management, business, or legal profession (21.7% vs. 9.4%, *p* < 0.001), and telework five or more days (16.9% vs. 5.3%, *p* < 0.001). Individuals who used artificial intelligence at work (mean = 7.1, SE = 0.1) had significantly higher life satisfaction scores than those who did not use artificial intelligence at work (mean = 6.7, SE = 0.1; *p* = 0.010).

### 3.3 Weighted descriptive statistics on artificial intelligence task use at work

The most common artificial intelligence task was “searching for information or technical help” (35.8%, 95% CI: 32.1%, 39.5%), followed by “writing communications, documentation, or instructions” (31.6%, 95% CI: 28.4%, 34.9%), “generating ideas” (31.4%, 95% CI: 28.0%, 34.8%), and “interpreting/translating/summarizing” (30.1%, 95 CI: 26.9%, 33.3%; Table 2). The bottom three artificial intelligence tasks were “coding or modeling” (10.7%, 95% CI: 9.2%, 12.2%), “medical care” (5.9%, 95% CI: 3.9%, 7.9%), and “managing logistics or supply chains” (4.3%, 95% CI: 2.2%, 6.4%).

**Table 2:** Weighted prevalence of artificial intelligence task use and weighted distribution of artificial intelligence task count at work among employed U.S. adults, 2026 Household Trends and Outlook Pulse Survey. Weighted population = 151,990,638.

Panel A. Weighted prevalence of artificial intelligence task use.		
Artificial intelligence task domain	%	95% CI
Searching for information or technical help	35.8	(32.1, 39.5)
Writing communications, documentation, or instructions	31.6	(28.4, 34.9)
Generating ideas	31.4	(28.0, 34.8)
Interpreting/translating/summarizing	30.1	(26.9, 33.3)
Administrative tasks	26.7	(23.5, 30.0)
Data analysis and visualization	20.1	(17.6, 22.6)
Tutoring/training/educational assistance	15.1	(12.5, 17.6)
Customer support	11.3	(8.6, 14.0)
Coding or modeling	10.7	(9.2, 12.2)
Medical care	5.9	(3.9, 7.9)
Managing logistics or supply chains	4.3	(2.2, 6.4)


Panel B. Weighted distribution of artificial intelligence task count.		
Number of artificial intelligence tasks	%	95% CI
0	48.3	(44.1, 52.4)
1	9.1	(7.3, 11.0)
2	7.4	(6.0, 8.7)
3	7.4	(5.5, 9.3)
4	5.3	(4.5, 6.2)
5	5	(4.2, 5.9)
6	5.1	(4.2, 6.0)
7	4.5	(3.3, 5.7)
8	4.5	(2.1, 6.8)
9	1.9	(1.3, 2.6)
10	1.1	(0.5, 1.6)
11	0.4	(0.2, 0.6)

### 3.4 Multivariable results

Fig. 1 and Table 3, Table 4, Table 5 show the associations between extent of artificial intelligence use at work and psychosocial well-being among employed U.S. adults. Across all model specifications, greater extent of artificial intelligence use at work was significantly associated with greater life satisfaction (Table 3). Model 1 indicated that, without adjusting for covariates, a 1-SD increase in artificial intelligence task count was significantly associated with a 0.10 SD (95% CI: 0.05, 0.16, *p* < 0.001) increase in life satisfaction score. This association was sustained after adjusting for demographic characteristics in Model 2 (0.10 SD increase, 95% CI: 0.05, 0.15, *p* < 0.001), but slightly attenuated when additionally adjusting for SES and region of residence in Model 3 (0.08 SD increase, 95% CI: 0.03, 0.13, *p* = 0.003). Controlling for differences in occupation type and telework frequency in Model 4 strengthened this association to a 0.11 SD increase (95% CI: 0.06, 0.16, *p* < 0.001).

**Fig. 1:**
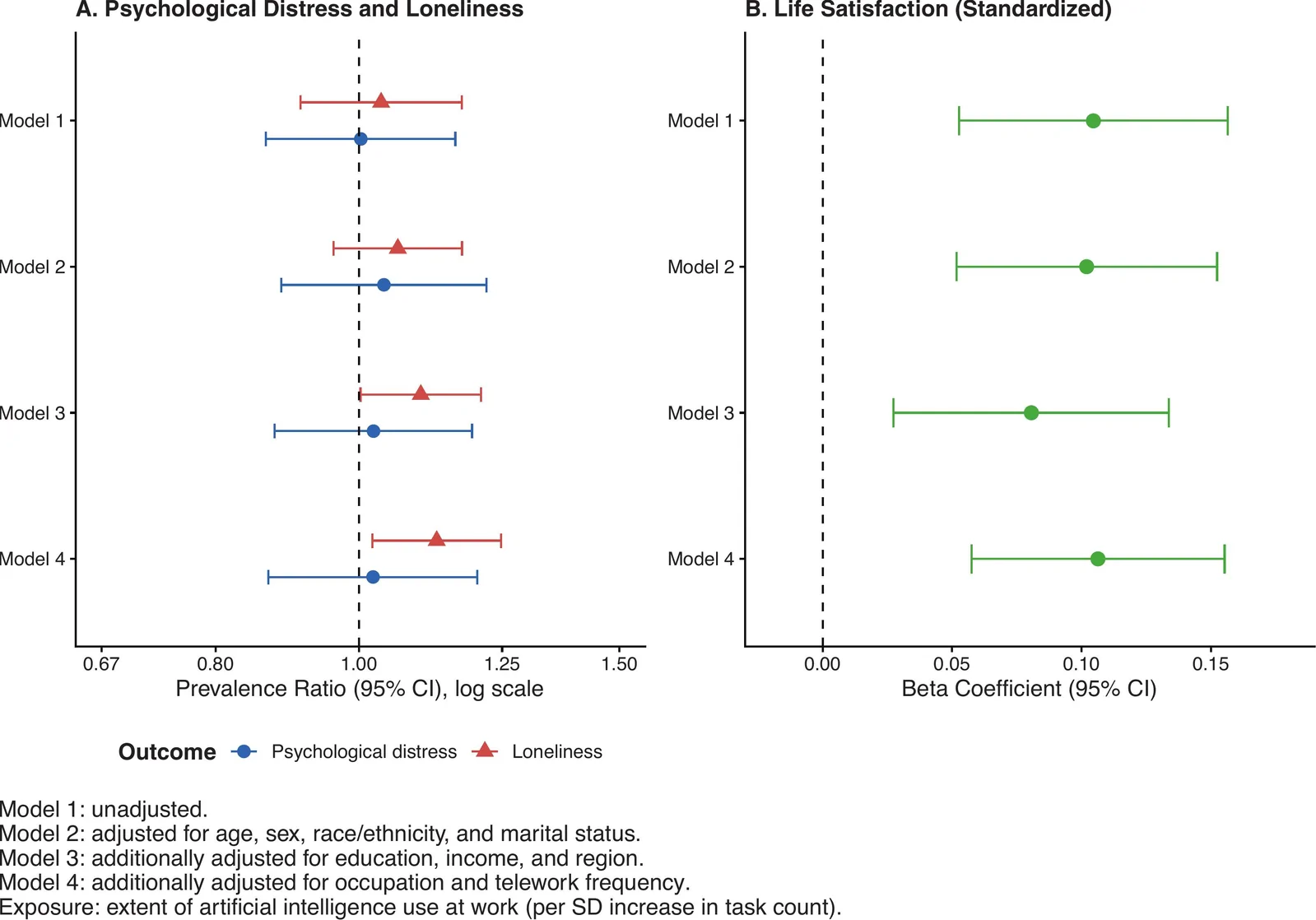
Associations between the extent of artificial intelligence use at work and psychosocial health among employed U.S. Adults, 2026 Household Trends and Outlook Pulse Survey. Weighted population = 151,990,638.

**Table 3:** Associations between the extent of artificial intelligence use at work and life satisfaction (standardized) among employed U.S. adults, 2026 Household Trends and Outlook Pulse Survey. Weighted population = 151,990,638.

	Model 1	Model 2	Model 3	Model 4
Characteristic	Beta (95% CI)	Beta (95% CI)	Beta (95% CI)	Beta (95% CI)
Artificial intelligence task count (SD)	0.10 (0.05, 0.16)	0.10 (0.05, 0.15)	0.08 (0.03, 0.13)	0.11 (0.06, 0.16)
Age		0.01 (0.01, 0.02)	0.01 (0.01, 0.02)	0.01 (0.01, 0.02)

Sex				
Male		–	–	–
Female		−0.01 (−0.15, 0.13)	−0.01 (−0.14, 0.13)	0.02 (−0.09, 0.14)

Race/ethnicity				
White		–	–	–
Hispanic		0.12 (−0.08, 0.32)	0.19 (−0.01, 0.40)	0.20 (−0.01, 0.40)
Black		−0.12 (−0.49, 0.26)	−0.10 (−0.46, 0.25)	−0.08 (−0.37, 0.22)
Asian		0.12 (−0.04, 0.27)	0.17 (0.02, 0.31)	0.20 (0.04, 0.35)
Other		0.04 (−0.21, 0.29)	0.13 (−0.12, 0.39)	0.13 (−0.11, 0.37)

Marital status				
Married		–	–	–
Divorced/Separated/Widowed		−0.18 (−0.35, 0.00)	−0.12 (−0.30, 0.06)	−0.12 (−0.30, 0.06)
Never married		−0.23 (−0.44, −0.02)	−0.16 (−0.38, 0.06)	−0.18 (−0.39, 0.03)

Educational attainment				
High school or less			–	–
Some college/Associate's degree			−0.14 (−0.36, 0.07)	−0.09 (−0.31, 0.13)
Bachelor's degree			0.02 (−0.18, 0.21)	0.10 (−0.12, 0.32)
Graduate degree			−0.04 (−0.25, 0.16)	0.02 (−0.20, 0.24)

Household income				
Less than $50,000			–	–
$50,000–$99,999			−0.10 (−0.34, 0.15)	−0.07 (−0.30, 0.15)
$100,000–$149,999			0.22 (−0.03, 0.46)	0.24 (0.00, 0.49)
$150,000 and above			0.16 (−0.07, 0.39)	0.20 (−0.04, 0.44)

Census region				
Northeast			–	–
Midwest			0.00 (−0.17, 0.16)	−0.02 (−0.18, 0.14)
South			0.08 (−0.08, 0.24)	0.07 (−0.10, 0.23)
West			−0.19 (−0.36, −0.02)	−0.20 (−0.37, −0.03)

Occupation				
Computer				–
Trades, Transport, and Farming				0.20 (−0.10, 0.49)
Education/Social Services/Arts				0.15 (−0.10, 0.39)
Engineering/Architecture/Sciences				0.13 (−0.10, 0.36)
Healthcare				0.16 (−0.08, 0.40)
Management/Business/Legal				0.09 (−0.11, 0.29)
Office/Administrative Support				−0.12 (−0.50, 0.27)
Sales				0.35 (0.08, 0.62)
Services/Security/Food/Personal Care				0.13 (−0.16, 0.42)

Telework frequency				
No telework				–
1–2 days				−0.22 (−0.47, 0.03)
3–4 days				−0.08 (−0.26, 0.09)
5+ days				−0.14 (−0.29, 0.01)

**Table 4:** Associations between the extent of artificial intelligence use at work and loneliness among employed U.S. adults, 2026 Household Trends and Outlook Pulse Survey. Weighted population = 151,990,638.

	Model 1	Model 2	Model 3	Model 4
Characteristic	PR (95% CI)	PR (95% CI)	PR (95% CI)	PR (95% CI)
Artificial intelligence task count (SD)	1.04 (0.91, 1.17)	1.06 (0.96, 1.17)	1.10 (1.01, 1.21)	1.13 (1.02, 1.25)
Age		0.98 (0.97, 0.99)	0.98 (0.98, 0.99)	0.99 (0.98, 0.99)

Sex				
Male		1.00	1.00	1.00
Female		1.22 (0.95, 1.56)	1.20 (0.96, 1.50)	1.15 (0.92, 1.46)

Race/ethnicity				
White		1.00	1.00	1.00
Hispanic		0.81 (0.56, 1.17)	0.72 (0.50, 1.03)	0.70 (0.50, 1.00)
Black		1.29 (0.89, 1.87)	1.21 (0.86, 1.72)	1.14 (0.85, 1.54)
Asian		1.01 (0.72, 1.44)	0.93 (0.67, 1.28)	0.90 (0.66, 1.23)
Other		0.79 (0.48, 1.31)	0.70 (0.43, 1.15)	0.72 (0.46, 1.14)

Marital status				
Married		1.00	1.00	1.00
Divorced/Separated/Widowed		1.63 (1.23, 2.17)	1.40 (1.04, 1.89)	1.43 (1.06, 1.92)
Never married		1.89 (1.40, 2.56)	1.67 (1.25, 2.24)	1.73 (1.32, 2.27)

Educational attainment				
High school or less			1.00	1.00
Some college/Associate's degree			0.86 (0.63, 1.17)	0.85 (0.62, 1.16)
Bachelor's degree			1.09 (0.79, 1.49)	1.08 (0.78, 1.49)
Graduate degree			1.09 (0.77, 1.53)	1.08 (0.75, 1.56)

Household income				
Less than $50,000			1.00	1.00
$50,000–$99,999			1.11 (0.80, 1.53)	1.10 (0.79, 1.52)
$100,000–$149,999			0.70 (0.48, 1.02)	0.70 (0.47, 1.04)
$150,000 and above			0.55 (0.35, 0.85)	0.58 (0.36, 0.91)

Census region				
Northeast			1.00	1.00
Midwest			1.08 (0.80, 1.44)	1.08 (0.81, 1.44)
South			1.06 (0.80, 1.41)	1.08 (0.82, 1.42)
West			1.37 (1.02, 1.85)	1.39 (1.04, 1.86)

Occupation				
Computer				1.00
Trades, Transport, and Farming				0.99 (0.55, 1.76)
Education/Social Services/Arts				0.99 (0.58, 1.69)
Engineering/Architecture/Sciences				0.91 (0.50, 1.67)
Healthcare				1.10 (0.64, 1.91)
Management/Business/Legal				0.77 (0.46, 1.28)
Office/Administrative Support				1.24 (0.75, 2.05)
Sales				0.82 (0.42, 1.60)
Services/Security/Food/Personal Care				1.09 (0.59, 2.01)

Telework frequency				
No telework				1.00
1–2 days				1.21 (0.91, 1.61)
3–4 days				0.70 (0.50, 0.98)
5+ days				0.90 (0.68, 1.19)

**Table 5:** Associations between the extent of artificial intelligence use at work and symptoms of moderate-to-severe psychological distress among employed U.S. adults, 2026 Household Trends and Outlook Pulse Survey. Weighted population = 151,990,638.

	Model 1	Model 2	Model 3	Model 4
Characteristic	PR (95% CI)	PR (95% CI)	PR (95% CI)	PR (95% CI)
Artificial intelligence task count (SD)	1.00 (0.87, 1.16)	1.04 (0.89, 1.22)	1.02 (0.88, 1.19)	1.02 (0.87, 1.20)
Age		1.00 (0.98, 1.01)	1.00 (0.98, 1.01)	1.0 (0.98, 1.01)

Sex				
Male		1.00	1.00	1.00
Female		1.48 (0.99, 2.23)	1.51 (1.02, 2.26)	1.72 (1.13, 2.60)

'Race/ethnicity				
White		1.00	1.00	1.00
Hispanic		0.67 (0.33, 1.37)	0.68 (0.33, 1.39)	0.64 (0.32, 1.31)
Black		0.85 (0.51, 1.42)	0.91 (0.54, 1.52)	0.89 (0.53, 1.48)
Asian		0.77 (0.36, 1.65)	0.66 (0.30, 1.45)	0.62 (0.28, 1.37)
Other		1.37 (0.73, 2.59)	1.30 (0.69, 2.47)	1.23 (0.69, 2.20)

Marital status				
Married		1.00	1.00	1.00
Divorced/Separated/Widowed		0.93 (0.53, 1.63)	0.89 (0.50, 1.61)	0.90 (0.50, 1.61)
Never married		1.75 (1.12, 2.71)	1.69 (1.06, 2.71)	1.72 (1.07, 2.77)

Educational attainment				
High school or less			1.00	1.00
Some college/Associate's degree			2.22 (1.15, 4.26)	2.33 (1.21, 4.48)
Bachelor's degree			2.07 (1.06, 4.06)	2.15 (1.05, 4.37)
Graduate degree			1.84 (0.92, 3.68)	2.12 (1.01, 4.43)

Household income				
Less than $50,000			1.00	1.00
$50,000–$99,999			1.09 (0.60, 1.98)	1.14 (0.62, 2.10)
$100,000–$149,999			0.74 (0.37, 1.45)	0.75 (0.37, 1.53)
$150,000 and above			0.77 (0.38, 1.56)	0.80 (0.36, 1.75)

Census region				
Northeast			1.00	1.00
Midwest			1.25 (0.68, 2.30)	1.20 (0.67, 2.17)
South			0.82 (0.48, 1.42)	0.82 (0.48, 1.43)
West			1.37 (0.81, 2.31)	1.39 (0.82, 2.35)

Occupation				
Computer				1.00
Trades, Transport, and Farming				0.70 (0.24, 2.01)
Education/Social Services/Arts				0.54 (0.23, 1.27)
Engineering/Architecture/Sciences				0.95 (0.33, 2.68)
Healthcare				0.48 (0.19, 1.22)
Management/Business/Legal				0.85 (0.38, 1.92)
Office/Administrative Support				0.89 (0.33, 2.39)
Sales				0.44 (0.15, 1.30)
Services/Security/Food/Personal Care				1.00 (0.37, 2.70)

Telework frequency				
No telework				1.00
1–2 days				0.95 (0.56, 1.61)
3–4 days				0.41 (0.22, 0.77)
5+ days				0.76 (0.45, 1.28)

We observed a positive association between extent of artificial intelligence use at work and loneliness (Table 4). There was a non-significant association in Model 1 (prevalence ratio [PR] = 1.04, 95% CI: 0.91, 1.17, *p* = 0.59), which remained non-significant but strengthened in Model 2 when adjusting for demographic characteristics (adjusted PR [aPR] = 1.06, 95% CI: 0.96, 1.17, *p* = 0.23). However, incorporating SES and region of residence into Model 3 strengthened the association to statistical significance (aPR = 1.10, 95% CI: 1.01, 1.21, *p* = 0.044), indicating negative confounding by SES and region of residence. This association's magnitude strengthened in Model 4 when additionally adjusting for occupation type and telework frequency (aPR = 1.13, 95% CI: 1.02, 1.25, *p* = 0.018). There was no evidence of a statistically significant association between extent of artificial intelligence use at work and symptoms of moderate-to-severe psychological distress across all four models (all *p* ≥ 0.63; Table 5).

## 4 Discussion

We examined associations between artificial intelligence use at work and psychosocial well-being outcomes among a nationally representative sample of employed U.S. adults. Consistent with the hypothesized productivity pathway, greater artificial intelligence use at work was significantly associated with higher life satisfaction after adjusting for demographic characteristics, SES, region of residence, occupation type, and telework frequency. Consistent with the hypothesized interpersonal-displacement pathway, greater artificial intelligence use at work was also significantly associated with a higher prevalence of feeling lonely. We found no evidence of an association between artificial intelligence use at work and moderate-to-severe psychological distress, which does not support the hypothesized work-intensification pathway and is consistent with null findings from a recent target trial emulation (Perlis et al., 2026a). Collectively, these findings add to the growing literature on artificial intelligence use and health (Perlis et al., 2026a, Perlis et al., 2026b) and provide evidence to inform future longitudinal studies.

The association between greater artificial intelligence use at work and both higher life satisfaction and increased loneliness may appear contradictory, though the two outcomes were moderately correlated with one another (*r* = −0.36), as were psychological distress and life satisfaction (*r* = −0.46) and psychological distress and loneliness (*r* = 0.45). These moderate correlations suggest that the three outcomes, while related, capture distinct dimensions of psychosocial well-being. Returning to the loneliness finding specifically, one possible explanation is that artificial intelligence users are more likely to work remotely, and it is remote work, rather than artificial intelligence use, that drives the association with loneliness. To assess whether remote work explains this association, we additionally adjusted for telework frequency in the final model. The association between artificial intelligence use at work and loneliness was strengthened when adjusting for telework frequency. This pattern is more consistent with a broader displacement of informal interpersonal workplace exchange that is not captured by telework status alone. Since the present study did not measure workplace social interaction patterns or work intensity, further research with more granular measures is needed to confirm this mechanism.

This study has several limitations. First, given the cross-sectional design, the observed associations cannot be interpreted as causal. Reverse causation is plausible. For example, individuals experiencing greater loneliness may withdraw from in-person collaboration and rely more on artificial intelligence tools to complete work tasks, rather than artificial intelligence use itself increasing loneliness. Second, although the present analysis adjusted for a comprehensive set of covariates, unmeasured confounding cannot be ruled out. Longitudinal studies are needed to establish temporality and evaluate whether the extent of artificial intelligence use at work has a causal effect on psychosocial wellbeing.

## 5 Conclusions

The results from this study of employed U.S. adults suggest that artificial intelligence use at work is associated with both life satisfaction and loneliness, but not with overall psychological distress. The findings are consistent with the idea that artificial intelligence use at work may enhance productivity and reduce interactions with other workers, but may not lead to work intensification and fatigue. Life satisfaction, loneliness, and psychological distress are related concepts, but seem to capture different dimensions of psychosocial well-being. The loneliness finding is consistent with, though does not demonstrate, a broader displacement of informal interpersonal workplace exchange associated with artificial intelligence use at work that goes beyond telework. Because productivity, interpersonal displacement, and work intensification were not measured in this study, this mechanism should be treated as a plausible interpretation rather than an established pathway. Longitudinal studies incorporating direct measures of workplace social interaction and work intensity are needed to test this interpretation, and to clarify the precise relationship between increasing artificial intelligence use at work and the psychosocial well-being of employed adults.

## CRediT authorship contribution statement

**Jemar R. Bather:** Writing – original draft, Visualization, Software, Project administration, Methodology, Investigation, Formal analysis, Conceptualization. **Adolfo G. Cuevas:** Writing – review & editing, Validation, Investigation, Conceptualization. **Melody S. Goodman:** Writing – review & editing, Validation, Investigation, Conceptualization. **José A. Pagán:** Writing – review & editing, Validation, Investigation, Conceptualization.

## Funding

This research did not receive any specific grant from funding agencies in the public, commercial, or not-for-profit sectors.

## Declaration of competing interest

The authors declare that they have no known competing financial interests or personal relationships that could have appeared to influence the work reported in this paper.

## Data Availability

The data supporting this study's findings are publicly available on the US Census Bureau website (https://www.census.gov/programs-surveys/household-pulse-survey/data/datasets.html).
