# Supplementary material for: Associations of artificial intelligence use at work with psychological distress, loneliness, and life satisfaction among employed US adults
Source: Prev Med Rep. 2026 Sep 18;71:103640. doi: 10.1016/j.pmedr.2026.103640 (PMC13634062; doi:10.1016/j.pmedr.2026.103640)
Supplement: Supplementary file 1 [file mmc1.docx]

**Supplemental Table 1.** Weighted correlations between outcome measures among employed U.S. adults, 2026 Household Trends and Outlook Pulse Survey. Weighted population = 151,990,638.

| **Outcome Pair** | **r** | **95% CI** |
| --- | --- | --- |
| PHQ-4 total score & Life satisfaction | -0.46 | (-0.53, -0.39) |
| PHQ-4 total score & Loneliness | 0.45 | (0.39, 0.52) |
| Life satisfaction & Loneliness | -0.36 | (-0.43, -0.29) |
| Abbreviation: PHQ = Patient Health Questionnaire  Point-biserial correlation reported for pairs involving loneliness (binary). Variance estimated using successive difference replication weights (R = 80); Confidence intervals based on the Fisher z transformation. | | |
